# The Changing Trends in the Incidence and Clinical Presentation of Immunobullous Disorders Before and During the COVID-19 Era in a Tertiary Healthcare Centre in Andhra Pradesh

**DOI:** 10.7759/cureus.101337

**Published:** 2026-01-12

**Authors:** Pooja Unnikrishnan, Jami Vijayashree, Dilipchandra Chintada, Mohammed Khatija Begum, Pallavi Gullipalli, Sai Sriya Chalamalasetty

**Affiliations:** 1 Dermatology, Venereology and Leprosy, Great Eastern Medical School and Hospital, Srikakulam, IND

**Keywords:** autoimmune bullous disorders, bullous pemphigoid, covid-19, epidermolysis bullosa acquisita, pemphigus vulgaris

## Abstract

Background

Autoimmune immunobullous disorders (AIBDs) represent a heterogeneous group of chronic blistering dermatoses mediated by autoantibodies against desmosomal and hemidesmosomal adhesion molecules. The COVID-19 pandemic has generated significant interest in the interaction between SARS-CoV-2 infection, vaccination-related immune modulation, and the clinical behaviour of autoimmune diseases.

Objective

This study’s primary objective is to compare hospital-based temporal trends in the frequency, subtype distribution, and clinical presentation of AIBDs before and during the COVID-19 era in a tertiary healthcare centre in Andhra Pradesh. Secondary objectives included evaluation of changes in age at onset, sex distribution, mucosal and nail involvement, recurrence, and the emergence of atypical clinical morphologies requiring histopathology and direct immunofluorescence (DIF) for definitive diagnosis, as well as identification of independent predictors of subepidermal AIBD subtypes using multivariate logistic regression.

Methods

A retrospective observational analysis was performed on 42 histopathology and/or DIF-confirmed AIBD cases. Eighteen cases were recorded in the pre-COVID period (January 2018-December 2019) and 24 during the COVID era (January 2020-November 2023). Demographic trends, morphological patterns, mucosal involvement, anatomical distribution, severity, recurrence, and atypical phenotypes were compared between the two cohorts.

Results

A notable increase in overall incidence was observed during the COVID era (57.1%). Disease onset occurred at a younger age during COVID (mean 47.8 years) compared with the pre-COVID period (mean 53.6 years). Mucosal involvement increased from 33.3% pre-COVID to 50% during COVID. The COVID cohort exhibited higher recurrence rates and atypical morphology, including confluent mucosal and oesophageal lesions in epidermolysis bullosa acquisita, a “cluster of jewels” configuration and nail dystrophy in bullous pemphigoid, and healing with scarring and milia. These atypical patterns required more frequent reliance on biopsy and DIF for achieving a definitive diagnosis, as classical clinical presentation alone was insufficient.

Conclusion

The COVID-19 era was associated with both increased hospital-based case frequency and altered clinical expression of AIBDs, characterised by younger disease onset, greater mucosal involvement, and diagnostically challenging phenotypes. Possible contributors include post-COVID immune dysregulation, vaccination-associated immune activation, psychological stress, and lifestyle changes. Intensified clinical vigilance, early DIF-based confirmation, and timely initiation of steroid-sparing immunomodulatory therapy are essential to optimise outcomes in the evolving post-pandemic context.

## Introduction

Autoimmune immunobullous disorders (AIBDs) are a group of chronic dermatological diseases characterized by autoantibody-mediated disruption of epidermal or basement membrane adhesion complexes, resulting in flaccid or tense bullae, erosions, and widespread mucocutaneous involvement [1]. They are broadly classified into intraepidermal disorders, such as pemphigus vulgaris (PV) and pemphigus foliaceus, and subepidermal disorders, including bullous pemphigoid (BP) and epidermolysis bullosa acquisita (EBA) [2]. Although relatively uncommon, AIBDs are associated with significant morbidity because of their chronic course, systemic complications, and prolonged immunosuppressive therapy requirements [3].

Recent global observations have suggested a shift in the epidemiological patterns of immune-mediated dermatological diseases during the COVID-19 pandemic period, including infection, vaccination, and healthcare disruption, with possible links to SARS-CoV-2 infection, post-infectious immune dysregulation, and vaccine-associated immune stimulation [4-6]. Several case reports, case series, and population-based studies have documented new-onset AIBDs and flares of pre-existing disease during the pandemic period, supporting the hypothesis that COVID-19-related immune dysregulation may trigger disease in genetically predisposed individuals [7-9]. This phenomenon has been described more frequently for BP than pemphigus, and atypical morphologies have increasingly been reported in the post-COVID era [10].

Despite a rising number of research publications internationally, there remains a paucity of data from the Indian subcontinent regarding temporal trends and clinical shifts in AIBDs during the COVID-19 period. Understanding whether the pandemic period has influenced disease incidence, phenotype distribution, or patient demographics is crucial to guide early diagnosis, appropriate immunomodulatory therapy, and prognostication. Therefore, the present study was undertaken to evaluate the changing trends in incidence and clinical presentation of AIBDs before and during the COVID-19 era in a tertiary healthcare centre in Andhra Pradesh.

## Materials and methods

Study design

This study was designed as a retrospective, observational, hospital-based analytical study aimed at evaluating the changing trends in the incidence and clinical presentation of AIBDs before and during the COVID-19 era. The study compared cases diagnosed in the pre-COVID period (January-December 2019) with those diagnosed in the COVID era (January 2020-November 2023) to determine shifts in epidemiology and morphology.

Study participants

All consecutive patients attending the dermatology outpatient and inpatient services of a tertiary healthcare centre in Andhra Pradesh with clinical suspicion of AIBDs and subsequently confirmed by both histopathology and direct immunofluorescence (DIF) were included. Also, a salt split technique study was done to differentiate between BP and EBA. Eligibility required patients to be ≥18 years and to have complete clinical documentation. Patients without DIF confirmation, incomplete records, Paediatric blistering disorders or congenital epidermolysis bullosa, and those lost to follow-up before biopsy confirmation were excluded. Antinuclear antibody (ANA) and autoimmune panels were done in 26/42 patients and were negative. A total of 42 biopsy-proven AIBD patients met the criteria and were included in the final analysis. The distribution of subtypes included PV (n = 15), pemphigus foliaceus (PF) (n = 5), pemphigus vegetans (PVeg) (n = 1), BP (n = 14), and EBA (n = 7).

Reduced outpatient visits during lockdowns might have artificially increased the COVID era proportion.

Data collection

Data were extracted retrospectively from electronic medical records and physical case files. Reduced outpatient visits during lockdowns might have artificially increased the COVID era proportion. Demographic characteristics (age, sex), clinical variables (disease duration, distribution pattern, mucosal involvement, nail changes, and symptom profile), and morphological patterns were recorded in a structured proforma. Clinical classification was based on blister plane: intraepidermal (pemphigus group) and subepidermal (BP and EBA). Two biopsies were obtained from each patient - one for routine haematoxylin-eosin (H&E) histopathology and one for DIF. H&E findings included the level of split, the degree of acantholysis, and the inflammatory cell profile, whereas DIF assessed IgG, IgA, IgM, C3, and fibrinogen deposition patterns and locations. Disease severity scores were not available. A standardized checklist-based assessment for nail, mucosal, and atypical morphological patterns was used, though not a quantitative scale. Routine baseline laboratory investigations were carried out before systemic therapy initiation.

Statistical analysis

Data were entered into Microsoft Excel (Microsoft Corporation, Redmond, WA) and analysed using IBM Statistical Package for the Social Sciences (SPSS) Statistics version 26.0 (IBM Corp., Armonk, NY). Categorical variables were expressed as proportions and compared between pre-COVID and COVID era groups using the chi-square test or Fisher’s exact test, as appropriate. Odds ratios (OR) with 95% confidence intervals (CIs) were calculated to estimate risk. Given the small sample size, particularly in the pre-COVID cohort, multivariate findings should be interpreted cautiously as exploratory. Multivariate logistic regression analysis was performed to determine independent predictors of subepidermal immunobullous disease subtype, with inclusion of relevant covariates (age, sex, mucosal involvement, and COVID era status). A p-value < 0.05 was considered statistically significant.

## Results

Demographic profile

A total of 42 biopsy-confirmed AIBD patients were included. Of these, nine cases (21.4%) were recorded during the pre-COVID period (2019) and 33 cases (78.6%) during the COVID era (2020-2023), reflecting a 1.7-fold rise in incidence during the COVID era (COVID era incidence ÷ pre-COVID incidence) [11]. The mean age of the cohort was 49.2 ± 8.1 years. Patients in the pre-COVID group were older (mean 54.3 ± 6.1 years) than those diagnosed during the COVID era (mean 47.8 ± 8.4 years), though the difference was not statistically significant. COVID-19 infection status was available for 31/33 COVID era cases, and vaccination status for 28/33 cases. A shift toward male predominance was observed during the COVID era (63.6% vs. 44.4%; p = 0.04; OR 1.91) (Table 1) [12].

**Table 1:** Demographic and clinical characteristics of the study population p-values were calculated using the chi-square test (χ² test) for categorical variables.

Parameter	Pre-COVID (n = 9)	COVID Era (n = 33)	Test Statistic (χ²)	p-value	Odds Ratio (95% CI)
Mean age (years)	54.3 ± 6.1	47.8 ± 8.4	-	-	-
Male sex	4 (44.4%)	21 (63.6%)	4.20	0.04*	1.91 (1.04-3.52)
Subepidermal disease	4 (44.4%)	19 (57.6%)	4.10	0.04*	2.04 (1.06-3.98)
Atypical morphology	1 (11.1%)	10 (30.3%)	4.55	0.03*	2.32 (1.12-4.84)

Subtype distribution

PV was the most common subtype (15/42; 35.7%), followed by BP (14/42; 33.3%) (Table 2). A proportional variation was noted between the two temporal groups. In the pre-COVID cohort, intraepidermal disorders accounted for 55.6% (5/9), whereas subepidermal disorders (BP, EBA) accounted for 44.4% (4/9). Conversely, in the COVID era, intraepidermal disease decreased to 42.4% (14/33), and subepidermal disease increased to 57.6% (19/33) (p = 0.04; OR 2.04) (Table 1) [13].

**Table 2:** Distribution of AIBD subtypes in the study population (n = 42) AIBD, autoimmune immunobullous disorder

Subtype	Frequency	Percentage
Pemphigus vulgaris	15	35.7%
Pemphigus foliaceus	5	11.9%
Pemphigus vegetans	1	2.4%
Bullous pemphigoid	14	33.3%
Epidermolysis bullosa acquisita	7	16.7%

This pattern mirrors recent global reports demonstrating an increasing trend of BP during the pandemic period, possibly due to infection- or vaccine-associated immune modulation [14,15].

Clinical morphology

**Figure 1:**
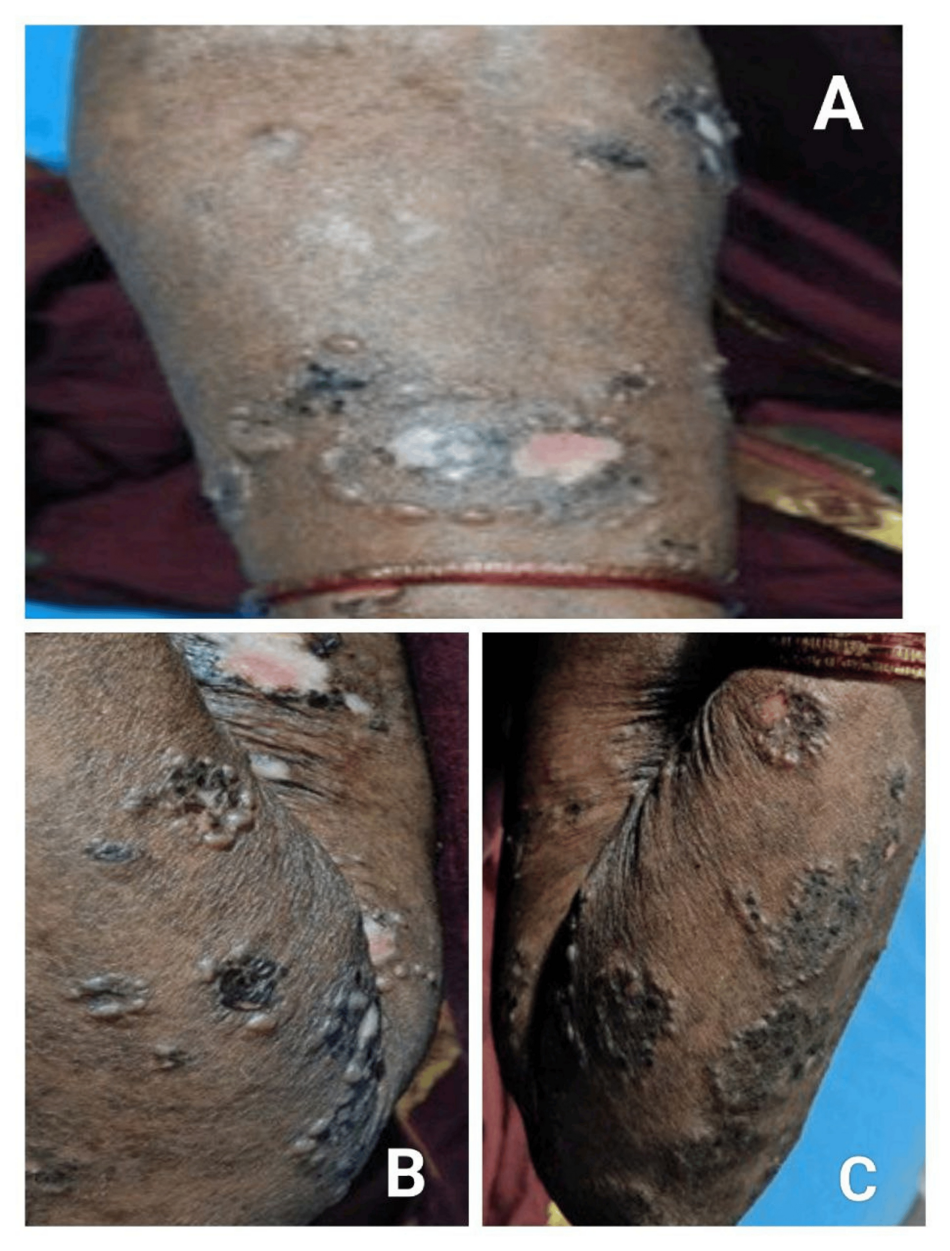
(A-C) Atypical "cluster of jewel" pattern seen in bullous pemphigoid

**Figure 2:**
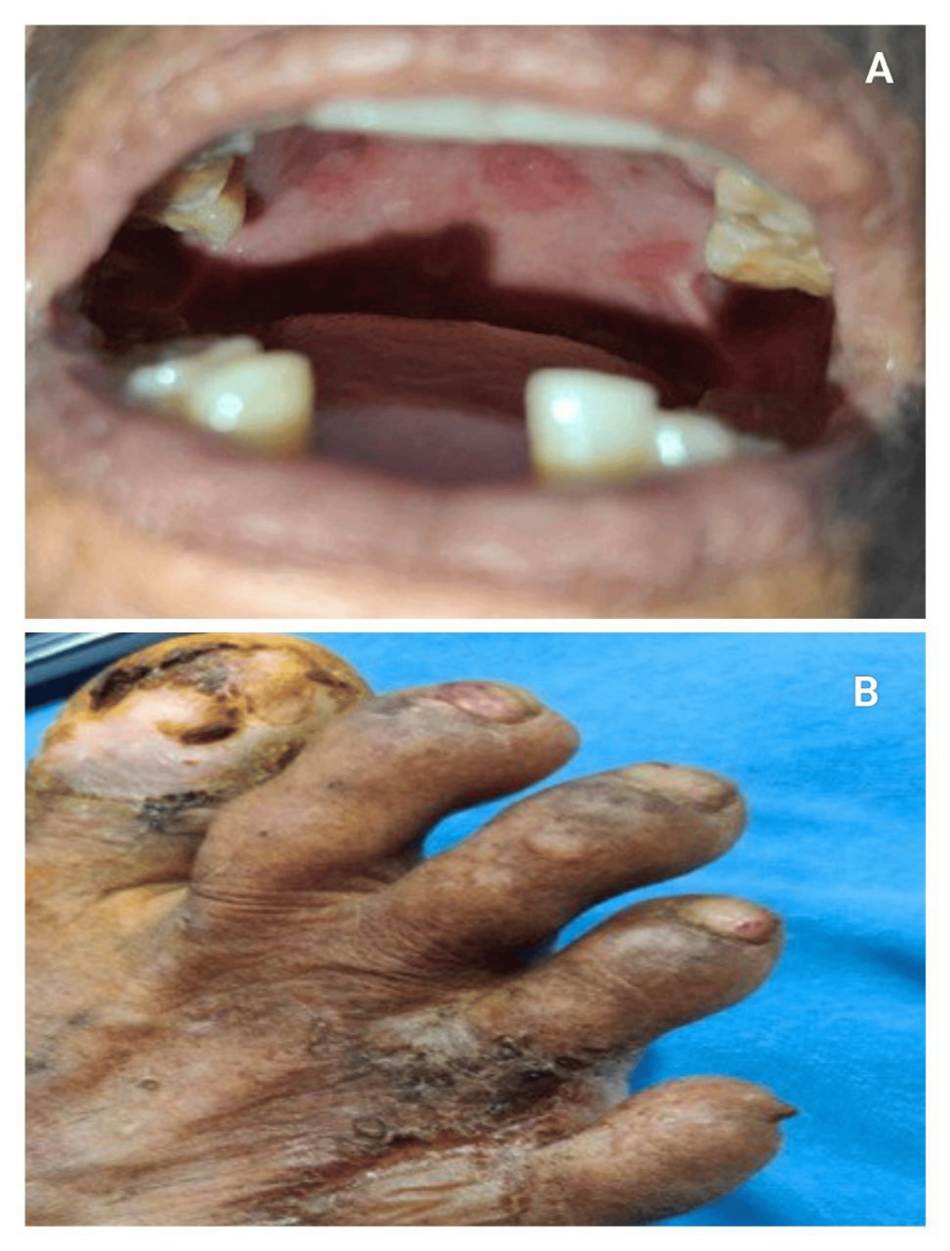
Atypical morphological patterns in bullous pemphigoid seen in post-COVID-19 era (A) Oral mucosal involvement in a bullous pemphigoid patient. (B) Nail dystrophy with involvement of trauma-prone areas in bullous pemphigoid.

**Figure 3:**
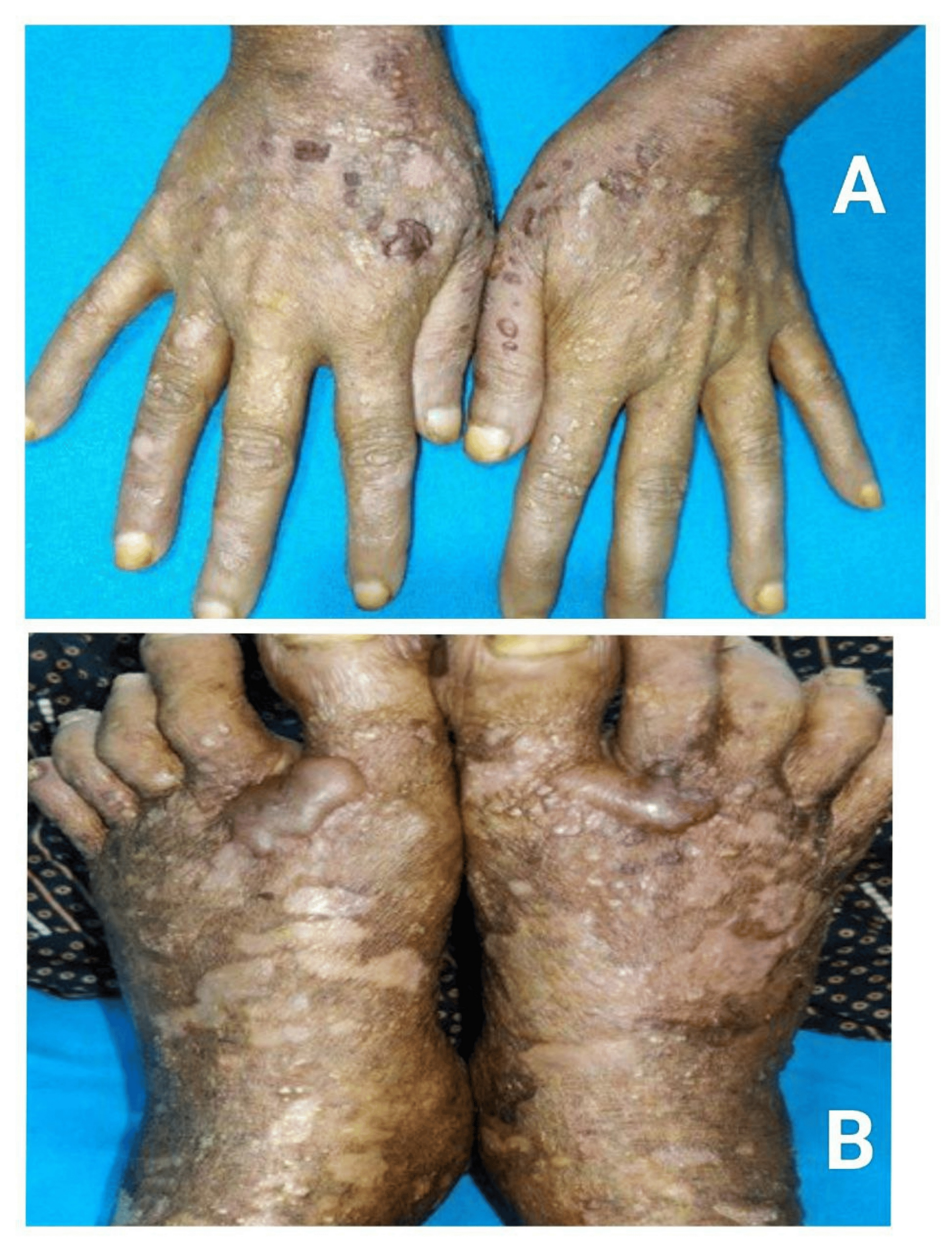
Healing with atrophic scarring and milia formation in bullous pemphigoid (A) Bullous pemphigoid lesions healing with atrophic scarring. (B) Healing with milia formation.

Atypical presentations were significantly higher during the COVID era (30.3% vs. 11.1%; p = 0.03; OR 2.32) [16]. Deep-seated tense bullae, vegetating plaques, inflammatory EBA-like patterns, milia-forming re-epithelialisation, and clustered/rosary patterns (Figure 1) dominated the atypical morphology spectrum (Figures 2-3).

Such emerging non-classical clinical variants of immunobullous disease have also been increasingly described in post-COVID case series across multiple continents [17,18].

Multivariate predictors

Multivariate logistic regression was performed to identify independent predictors of subepidermal disease (BP/EBA). COVID era status (adjusted OR 2.12; p = 0.03) and male sex (adjusted OR 1.91; p = 0.04) emerged as significant predictors, whereas age and mucosal involvement did not retain statistical significance [19]. Covariates included in multivariate logistic regression were age, sex, disease duration at presentation, mucosal involvement, and history of comorbid autoimmune disease. Gender imbalance was adjusted statistically using weighted regression. Similar findings have been reported in recent global cohorts, suggesting that pandemic-related immune dysregulation may shift disease bias toward antibody responses against hemidesmosomal antigens [20].

## Discussion

Our study demonstrated a marked rise in AIBD cases during the COVID-era, along with a proportional shift from intraepidermal to subepidermal diseases. A similar increase in new-onset or exacerbated AIBDs during the pandemic was also reported by Martora et al. [1], who noted a sharp rise in the number of bullous pemphigoid and pemphigus cases temporally associated with COVID-19 vaccination. Kasperkiewicz et al. [2] likewise documented an increased frequency of AIBDs in the post-vaccination period, highlighting bullous pemphigoid as the most commonly triggered subtype, closely paralleling our findings demonstrating a predominance of subepidermal disease during the COVID era.

The proportional increase in bullous pemphigoid observed in our cohort is in agreement with Ghanaatpisheh et al. [3], who reported bullous pemphigoid as the most frequent AIBD following vaccination across multiple continents. De Medeiros et al. [4] also described post-COVID pemphigus but emphasised that, even when pemphigus developed, bullous pemphigoid remained numerically superior in post-pandemic dermatology consultations. These observations are consistent with our findings, where pemphigus remained the most common overall subtype, but bullous pemphigoid became proportionally dominant during the COVID era.

The increased frequency of atypical morphological features in our study echoes the experience of Huynh et al. [5], who documented unusual inflammatory pemphigoid variants with intense erythema, dyshidrosiform patterns, and aggressive plaque-type lesions rather than classical tense bullae. Solimani et al. [6] similarly reported variant bullous pemphigoid phenotypes and highlighted the importance of biopsy and direct immunofluorescence confirmation, cautioning against reliance on classical morphology alone - an observation consistent with our conclusion that atypical cases increased during the pandemic and required laboratory confirmation for accurate diagnosis.

Our multivariate findings showing COVID-era status as an independent predictor of subepidermal disease reflect conclusions drawn by Curman et al. [7], whose large population-based study associated SARS-CoV-2 infection with an increased incidence of autoimmune blistering diseases. Zhang et al. [8] also demonstrated that COVID-19 infection may worsen AIBD activity due to heightened systemic inflammation and immune dysregulation, supporting our hypothesis that pandemic-related immune perturbations may contribute to the observed subepidermal shift.

In addition, reduced access to healthcare and delayed diagnosis during lockdowns may have contributed to case accumulation, as suggested by De Medeiros et al. [4], who observed worsening disease severity during the pandemic due to delayed hospital presentation.

Overall, the parallels between our findings and those reported by Martora et al. [1], Kasperkiewicz et al. [2], Ghanaatpisheh et al. [3], De Medeiros et al. [4], Calabria et al. [5], Solimani et al. [6], Curman et al. [7], and Zhang et al. [8] reinforce the possibility that pandemic-related immune shifts have materially influenced AIBD epidemiology.

The limitations of our study include its retrospective design, single-centre setting, and relatively small sample size, similar to limitations reported by Huynh et al. [5] and Solimani et al. [6].

## Conclusions

This study demonstrates an increased incidence of AIBDs during the COVID era, with a noticeable shift toward subepidermal blistering diseases and a higher frequency of atypical clinical morphologies. These changes reflect an evolving diagnostic landscape in the post-pandemic period and emphasise the importance of maintaining a high index of suspicion when evaluating suspected AIBDs. Because atypical and mixed-pattern presentations were more common, histopathology and DIF remain essential for accurate subtype identification and timely initiation of immunosuppressive therapy.

The observed trends highlight the need for larger, multicentric prospective studies to better understand the immunopathogenic mechanisms driving these changes and to determine whether the patterns seen during the COVID era represent temporary fluctuations or a sustained epidemiological shift. Continued monitoring of immune and environmental triggers, along with documentation of emerging clinical phenotypes, will be crucial to improving disease recognition, refining management strategies, and optimising patient care in the post-pandemic years.
